# Comment on Grabala et al. Radiological Outcomes of Magnetically Controlled Growing Rods for the Treatment of Children with Various Etiologies of Early-Onset Scoliosis—A Multicenter Study. *J. Clin. Med.* 2024, *13*, 1529

**DOI:** 10.3390/jcm13082434

**Published:** 2024-04-22

**Authors:** Casper S. Tabeling, Justin V. C. Lemans, Moyo C. Kruyt

**Affiliations:** Department of Orthopaedic Surgery, University Medical Center Utrecht, 3584 CX Utrecht, The Netherlands; j.v.c.lemans-3@umcutrecht.nl (J.V.C.L.); m.c.kruyt@umcutrecht.nl (M.C.K.)

We read with great interest the study titled “Radiological Outcomes of Magnetically Controlled Growing Rods for the Treatment of Children with Various Etiologies of Early-Onset Scoliosis—A Multicenter Study” by Grabala and colleagues [1]. The authors present the long-term treatment results of 161 early-onset scoliosis (EOS) patients treated with magnetically controlled growing rods (MCGR).

The authors report that “Growth in the T1–T12 and T1–S1 segments was calculated as the latest postoperative value minus the first postoperative value”. This same method was used to determine maintenance of curve correction. Figure 3 [1] illustrates that following MCGR implantation, the main curve reduced from 86° to 47° and was further reduced to 46° at the latest follow-up. However, it is unlikely that the main curve reduces during growth-friendly treatment. In fact, regarding the true performance of distraction-based implants, a decline of correction is usually observed [2,3,4,5,6].

A logical explanation for the reported findings may be that the last follow-up radiographs included the radiographs of the patients that underwent final fusion. Indeed, 48 out of the 161 had PSF that, according to the authors, contributed considerably to the curve correction and growth. The authors report an additional curve correction during fusion from 49° to 25°. Therefore, it seems that the radiographs after final fusion were included to determine the performance of MCGR, which would overestimate the corrective effect of the MCGR, both in curve correction and growth.

Although the results after final fusion are important, they should not be combined with the results of patients that are still treated with the growth-friendly technique because, based on the literature, this final fusion procedure accounts for 24% of the reported “growth” [5]. This can be seen in the authors’ own data as the final fusion increased T1–S1 height by 34 mm in those patients undergoing PSF. As a consequence, the numbers of the current study cannot be interpreted. To allow for comparison between growth-friendly implants, it is very important to report the “true” growth and curve maintenance, i.e., during the treatment with the device.

We would like to encourage the authors to present the results of only the patients who did not receive final fusion or, even better, we encourage the authors to present the results of patients who did not undergo final fusion. Better yet, include the results of the entire group with radiographs taken before final fusion as the last follow-up. These data would tremendously help the growing spine community to appreciate the value of the investigated MCGR device for the various etiologies of EOS.
